# Anatomage Table Vet for Teaching the Triadan Dental Classification System: A Brief Trial and Feedback from the Students

**DOI:** 10.3390/vetsci12121142

**Published:** 2025-11-29

**Authors:** Ginevra Merluzzi, Francesca Mercati, Elvio Lepri, Andrea Verini Supplizi, Cecilia Dall’Aglio

**Affiliations:** Department of Veterinary Medicine, University of Perugia, 06126 Perugia, Italy; ginevra.merluzzi@dottorandi.unipg.it (G.M.); elvio.lepri@unipg.it (E.L.); andrea.verini@unipg.it (A.V.S.); cecilia.dallaglio@unipg.it (C.D.)

**Keywords:** veterinary anatomy, innovative didactic, digital tools, 3D anatomy

## Abstract

Veterinary anatomy is essential in veterinary education and has traditionally been taught through lectures and cadaveric dissection. Recently, digital tools such as the Anatomage Table Vet (ATV) have emerged, offering new ways to visualize anatomical structures and enhance visuospatial skills. This study was conducted during the students’ first experience with the ATV and aimed to evaluate its effectiveness compared to traditional textbook-based methods for teaching the Triadan Dental Classification System (TDCS), as well as to gather feedback on its usability. Eighty-nine students attended a seminar on the ATV and completed a pre-test and post-test consisting of Likert-scale statements and theoretical questions. Students were divided into two groups: an ATV group and a textbook group. Both groups significantly improved their post-test scores, with no significant difference between them. Students’ self-reported confidence with both the ATV and the TDCS also increased. Feedback was generally positive, except for the ATV’s ease of use: most students found the tool stimulating, reporting that they would use it during their independent study time. In this trial, ATV use did not outperform textbooks. However, students reported high satisfaction with the teaching tool. Further research is recommended to understand how to effectively integrate it into the veterinary curriculum.

## Simple Summary

New technology is implemented every year, and teaching methods are continually updated to incorporate these new resources into the curriculum of all disciplines, especially medical ones. The Anatomage Table Vet (ATV) is a virtual dissection tool with the potential to aid in teaching animal anatomy and training students’ visuo-spatial skills and it is just beginning to be implemented in universities. This study describes students’ first approach with the ATV, evaluating the effectiveness of this instrument compared to traditional teaching methods while also gathering feedback on its use and enjoyability from the students. The results show that students benefited from using the ATV when challenged with learning new theoretical anatomical knowledge, but no significant difference was found compared with using the textbook. Students’ feedback indicated an overall strong acceptance of the tool and interest in using it also during their personal study time, although most students did not consider the ATV easy to use. The implementation of this new technology into the veterinary anatomy curriculum could enhance student’s interest and curiosity when approaching this challenging subject, but additional research is needed to better understand how to standardize its use and maximize the potential of this new resource.

## 1. Introduction

Anatomy is one of the first challenging classes encountered by students across different degree programs: medical, veterinary, nursing, physiotherapy and biotechnology, just to name a few. A thorough understanding of this subject is fundamentally important for diagnostics, clinical practice, as well as for research purposes. It requires students to develop an in-depth understanding of both simple organs and systems, as well as more complex internal structures [1,2].

Cadaveric dissection has always been the bedrock of anatomical practice and study, offering essential insights into the structural anatomy of both animal and human bodies [3]. In recent years, however, cadaver availability has declined [4,5], considering increasing curricular demands, the growing number of students, and expanding training objectives. This issue is particularly relevant in veterinary medical, where students must study the anatomy of multiple species and compare them. Ensuring adequate hands-on practice on cadavers from a variety of animal species has therefore become increasingly challenging.

Spatial visualization is of fundamental importance in the study of veterinary anatomy. Students must learn the characteristics of individual anatomical structures and their spatial relationships with surrounding elements while also comparing these features across different species. Although textbooks and atlases provide high-quality two-dimensional images, they often fail to represent complex three-dimensional dynamics between organs and systems. Visuospatial ability is defined as the ability to mentally manipulate objects in three dimensions and is considered extremely important for medical and veterinary students, as well as for surgeons in training. The growing tendencies to introduce three-dimensional anatomical models and innovative teaching strategies stems from the need to train this and strengthen skill [6,7].

The efficacy of such teaching innovations and technologies in anatomy has been investigated in numerous studies, mainly in medical and dentistry programs, as well as in challenging anatomical and pathological subjects, such as veterinary neuroanatomy [8]. Although most studies show that students significantly prefer 3D technologies over traditional teaching methods, their greater efficacy has not been unanimously demonstrated [6]. An example of new 3D tool that can be implemented into modern teaching approaches is the Anatomage Table Vet (ATV). The Anatomage company (Anatomage, Inc. Santa Clara, CA, USA), founded in 2004, began as a radiology and dental software company and now specializes in general anatomy. Both the human and veterinary tables provide 3D anatomical models using the Invivo5 software to render images obtained by CT scans while also incorporating other diagnostic imaging instruments like the MRI, ultrasound, and X-ray machine [4,9,10,11].

The ATV allows users to visualize models on a large touchscreen (127 × 76 cm) and digitally dissect them. All components of the model can be added or removed layer by layer or structure by structure, enabling students to explore the three-dimensional anatomy of animal cadavers and facilitate the identification of structures. The ATV also includes an annotation system: when students interact with a specific anatomical structure, its name is displayed on the screen. Thus, although the tool is image-based, it provides clear labeling that supports the identification of organs, tissues, and anatomical landmarks [1,2,12,13].

The human Anatomage Table (AT) includes both male and female models, whereas the ATV currently is equipped with only the female model of both dog and cat. This instrument can be used individually or in small groups, making it suitable for both personal study and group instruction [10]. Beyond its role in education, the AT has been successfully used in the surgical and radiological field [14,15,16], and it holds significant potential for veterinary medicine as well. Currently, in veterinary applications, the ATV has been used by a research group to describe the normal anatomical features of the dentition and maxillofacial structures of healthy rabbits [17].

When introducing new teaching strategies into established curricula, it is essential to rigorously evaluate their effectiveness, as well as to assess student acceptance of the new tools [9]. Numerous studies have been conducted on the AT, with most reporting positive feedback from the students, though some noted technical issues and graphical limitations [5,10,13,18].

However, similar studies have not yet been conducted with the newly developed ATV.

Accordingly, the first aim of this study was to test the effectiveness of the ATV compared with traditional methods in teaching the Triadan Dental Classification System (TDCS) [19] to students at the University of Perugia. The second aim was to gather student feedback on the ATV’s perceived usefulness, ease of use, and overall enjoyability.

## 2. Materials and Methods

A series of presentation days on the use of the ATV was organized at the Department of Veterinary Medicine of the University of Perugia. Attendance was voluntary and open to all interested students; however, only those with no prior training in either the TDCS or the ATV were included in this study. The seminar consisted of an introduction to the ATV, providing students with the skills required to understand and use the device independently, followed by a brief practical session on learning the TDCS for the classification of the dog’s teeth.

Before the activities commenced, the study project was approved by the Bioethics Committee of the University of Perugia (Prot. No. 314952). An Informed Consent Document, detailing the study procedures, was provided to the students. To ensure privacy, participants were asked to select a five-digit alphanumeric identification code instead of providing their name.

### 2.1. Pre-Test

Eighty-nine students were first asked to fill in a pre-test form organized as follows: four statements (A1–A4) assessing their familiarity with the ATV and TDCS, as well as their confidence in identifying dog’s teeth (Table 1); and six quiz questions (Q1–Q6) covering theoretical knowledge of the TDCS and dog teeth anatomy (Table 2). The statements were rated using a 5-point Likert scale [20], while the quiz questions were answered freely by the students and subsequently graded on a binary “correct or incorrect” basis, with a maximum achievable score of 6/6 (100%).

### 2.2. Anatomage Table Vet Presentation and Training Session

After the pre-test, the ATV presentation introduced students the different features and tools available, explaining how to properly handle the models and their associated structures.

The training session took place after the seminar and the students were divided into two groups: the ATV group (A), and the textbook, or control, group (C).

**Figure 1:**
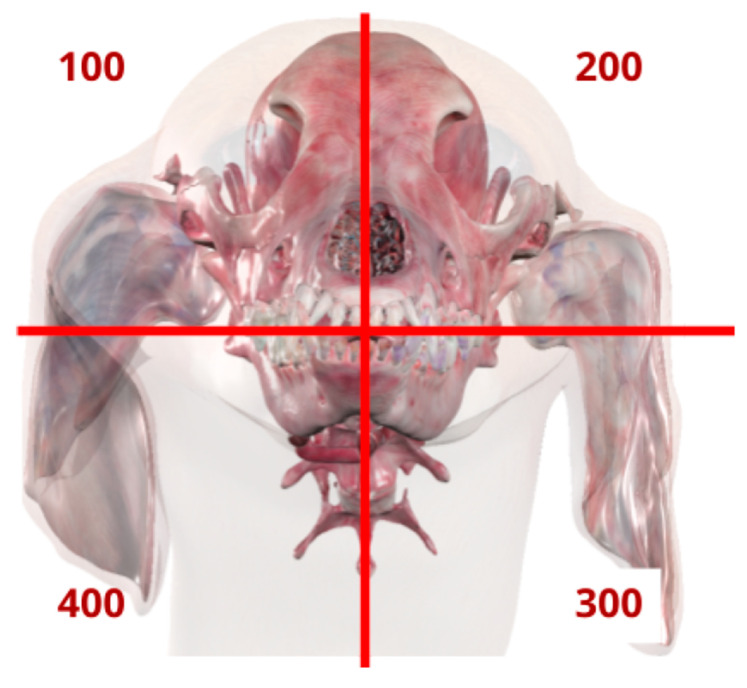
Excerpt from the Student’s Guide used by group A. The image shows a sample of the self-guided exercises included in the Student’s Guide, which was used by participants in group A to learn the TDCS.

For group A, the training activity “Canine Dental Triadan System” was downloaded from AnatomageShare (www.anatomageshare.com, accessed on 17 June 2024), and the necessary presets were uploaded to the University of Perugia’s ATV. Participants were given the Student’s Guide for the activity (Figure 1) and asked to complete the training exercise autonomously, using the presets uploaded onto the ATV. The activity consisted of five sections designed to teach students how to classify each tooth according to the TDCS while also offering an opportunity to review the canine dental formula.

**Figure 2:**
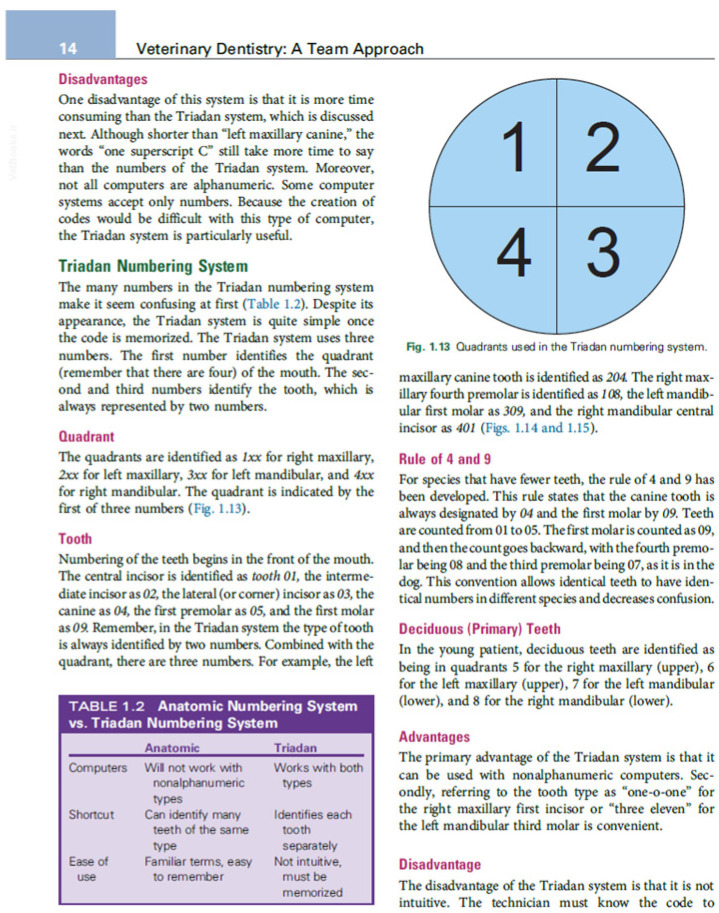
Excerpt from the textbook used by group C [19]. The image depicts a page from the textbook used for the training portion of group C, containing the theoretical information necessary for learning the TDCS protocol.

For group C, an excerpt from a textbook covering the same topics was provided to the students for an individual study session (Figure 2).

Both groups were given 15 min to review the assigned study materials.

### 2.3. Post-Test

Following the training session, participants completed the post-test, which included: two statements repeated from the pre-test (A3, A4); six feedback statements (F1–F6) regarding the perceived enjoyment and usefulness of the ATV (Table 3); and six quiz questions, four of which were comparable to the pre-test items (Q1–Q4), while two were identical to the pre-test questions (Q5, Q6).

Data were analyzed using commercially available statistical software (JASP version 0.16.1; The JASP Team, University of Amsterdam, Amsterdam, The Netherlands). A descriptive statistic was used for the answers to the test statements (A1–A4) and the feedback items on the ATV use (F1–F6). Data was tested for normality using the Shapiro–Wilk test and for homogeneity of variance using Levene’s test. Statistical tests were then applied as appropriate. A mixed-design ANOVA with status (pre-test vs. post-test) as a within-subjects factor and group (Anatomage vs. Control) as a between-subjects factor was conducted on quiz scores and level of confidence perceived by the students (answers to A3 and A4). Effect size was calculated using partial eta squared (η^2^). Significance was set at *p* < 0.05.

To evaluate the relationship between students’ perceptions and their performance, a correlation analysis was conducted between responses to the feedback questionnaire items (scored on a 0–5 Likert scale) and post-test quiz scores (0–6). Pearson correlation coefficients were calculated for variables considered continuous and normally distributed, while Spearman’s coefficients were also considered as a more conservative approach.

## 3. Results

### 3.1. Participants

Eighty-nine students took part in this study. Forty-seven students were assigned to group A (52.8%) and forty-two students to group C (47.2%).

### 3.2. Statements

In the pre-test, seventy-five students reported having no familiarity with the ATV, and eighty-five students had never heard of the TDCS. Overall, both groups provided significantly higher scores in the post-test statements (*p* < 0.01). The answers to the statements included in both the pre-test and post-test are shown in Table 4.

### 3.3. Quiz

**Figure 3:**
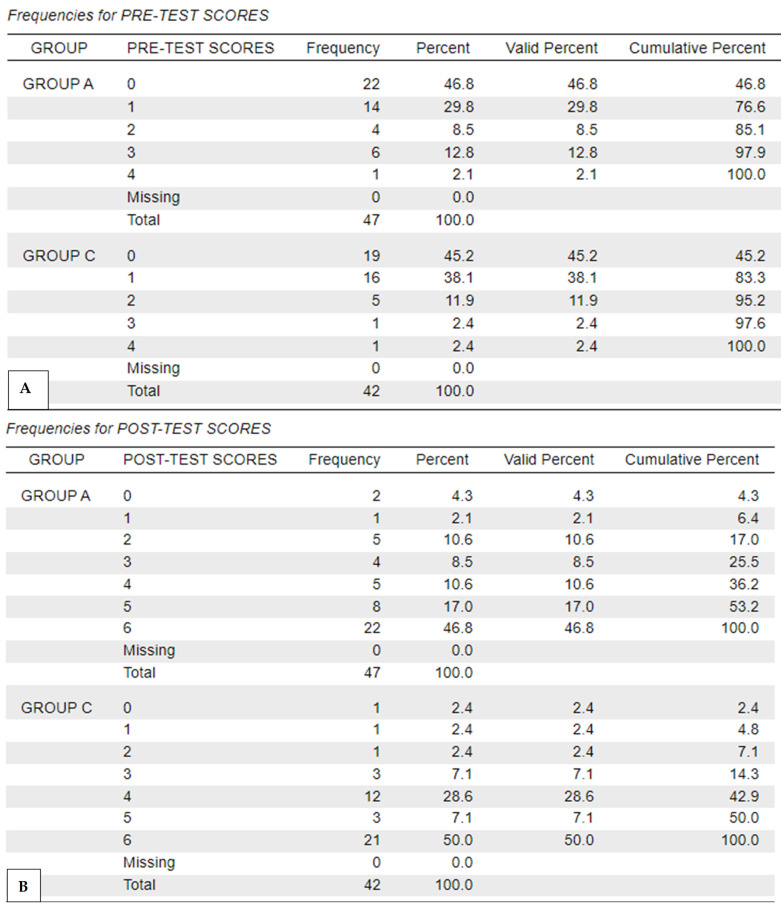
Frequencies for quiz scores (n/6) achieved in the pre-test (**A**) and post-test (**B**) by group A (Anatomage) and group C (Control).

Overall, both groups scored significantly higher in the post-test (*p* < 0.01), with no significant differences between groups. The frequencies for pre-test and post-test scores achieved by the two groups are shown in Figure 3.

**Figure 4:**
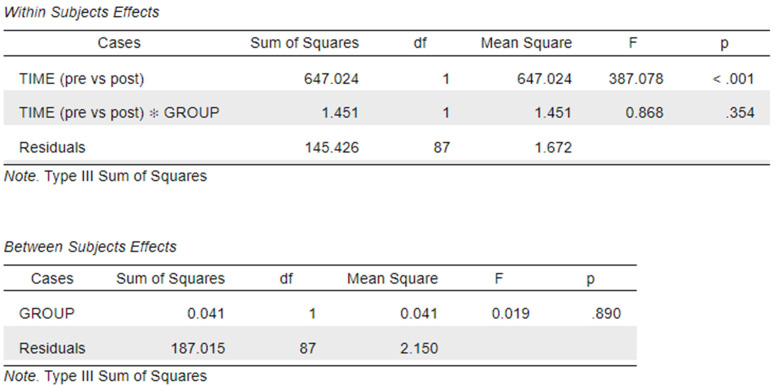
Mixed ANOVA analysis was performed to test the differences between pre-test and post-test scores (effect of time) and between groups (A vs. C) while looking for an interaction between time and group.

The mixed ANOVA analysis is shown in Figure 4. The interaction between time and group was not significant, indicating that the magnitude of improvement over time did not differ significantly between the two groups.

### 3.4. Feedback

Overall, eighty-three students agreed or strongly agreed that the ATV is useful for revising previously acquired anatomical knowledge, while seventy students agreed or strongly agreed that it is useful for acquiring new anatomical knowledge. Only fifty-five participants agreed or strongly agreed that the ATV was easy to use. Eighty-five students agreed or strongly agreed that using the ATV was stimulating, and eighty-three stated they would use the ATV during individual study time. No statistically significant differences were found between groups regarding overall feedback scores. The only feedback item that differed significantly between groups was F3:, forty-five students in group A versus thirty-one in group C considered the ATV useful for learning the TDCS (*p* < 0.01). The full answers given by the students to the feedback questions are shown in Table 5. The correlation analysis did not reveal any statistically significant relationships between students’ feedback responses and their post-test scores.

## 4. Discussion

Many studies have shown that the Anatomage Table (AT) is more effective when integrated into anatomical teaching curriculum without fully replacing other teaching methods, particularly cadaveric dissections [1,21,22,23,24,25,26]. For this reason, the AT is considered a valuable tool for anatomists, educators, and students; however, there is still no consensus that it should completely replace cadaver-based learning.

Furthermore, there is no unanimous agreement regarding the greater effectiveness of the AT compared with traditional methods in improving students’ performance. Some studies reported improved student performance after virtual dissections compared with cadaveric ones [27], whereas others found no significant differences in performance between students taught with the AT and those trained with cadaveric dissections when studying the musculoskeletal system [28].

The ATV includes an integrated annotation system: when students interact with a specific anatomical structure, its name appears on the screen in real time. This feature ensures that learners can immediately associate the visualized element with its correct terminology, reducing ambiguity and supporting accurate recognition. Although the tool is primarily image-based, the presence of dynamic labeling greatly enhances its educational value by guiding students within the virtual anatomical environment and reinforcing the association between visual information and anatomical nomenclature.

In addition to simple labeling, the annotation system contributes to a more structured learning experience by helping students identify organs, tissues, and anatomical landmarks without needing to consult external references. This seamless integration of visualization and labeling minimizes interruptions in the learning process and pro-motes continuous exploration. For novice learners, especially those encountering certain structures for the first time, such immediate feedback can be particularly beneficial, as it reduces cognitive load and prevents misinterpretation of complex images.

Moreover, the ability to highlight and label structures directly within the 3D environment allows students to better understand spatial relationships that might be difficult to infer from traditional 2D textbook figures. The system supports anatomical orientation by consistently providing contextual information that situates each structure within its broader functional and topographical framework.

Our study was conducted during the students’ first exposure to the Anatomage Table Vet (ATV) and, consequently, it reflects their initial and unconditioned experience with this innovative tool, capturing their spontaneous impressions and perceptions. This context is especially relevant for evaluating the students’ intuitive perception of the tool’s strengths and limitations before familiarity or training could influence their efficiency in its use. Importantly, our study did not aim to compare the effectiveness of the ATV with that of traditional cadaveric dissections, which are typically considered the gold standard for anatomical teaching in veterinary education. Instead, our objective was to assess the effectiveness of the ATV as an alternative to textbook-based learning in the context of teaching the Triadan Dental Classification System (TDCS). Focusing on this comparison allowed us to understand whether the ATV could offer a didactic advantage even for a topic that does not inherently require three-dimensional reasoning.

After the training session, both groups of students showed similar improvements in quiz scores. This outcome is likely attributable to the nature of the selected topic and the fact that the ATV group did not fully benefit from the instrument’s visuospatial capabilities. The TDCS, involving superficial structures and limited spatial complexity, does not require advanced visuospatial reasoning for its comprehension. As a result, students were able to learn the material effectively using either modality, and the expected advantage of a 3D visualization tool, like the ATV, did not emerge. The TDCS was intentionally chosen because it is not included in the standard veterinary anatomy curriculum, ensuring a uniform baseline of knowledge among participants. Moreover, its low anatomical complexity made it an appropriate topic for a first exploratory use of the ATV in a didactic setting, allowing us to isolate the general educational effects of introducing a new digital tool without the confounding effects of topic difficulty.

Although the TDCS does not strongly rely on advanced visuospatial skills, which are typically expected to benefit the most from 3D visualization environments, its selection enabled us to establish a controlled, accessible, and low-stress learning scenario. This framework offered an opportunity to evaluate how students react to and perceive the ATV when their cognitive load is minimal and not intensified by complex spatial reasoning demands. Understanding these baseline reactions is an essential preliminary step before investigating the potential of the ATV for more challenging anatomical topics, such as musculature, neuroanatomy, or relations among internal structures. Future iterations of this study could assess whether the didactic strengths of the ATV become more evident when students are required to interpret three-dimensional relationships that are difficult to visualize through two-dimensional textbook images.

All students who participated in the trial were eventually allowed to interact with the ATV regardless of group assignment and were subsequently asked to provide feedback on its perceived usefulness, ease of use, intuitiveness, and overall enjoyability. Collecting this feedback was essential to complement performance-based outcomes with subjective evaluations and to identify which aspects of the technology students found most supportive or motivating for their learning process.

In other previous studies, most of the students reported that the ATV made learning more engaging and enhanced their understanding of topographic anatomy [29]. Other research found that students preferred the ATV over textbooks and believed it made classroom teaching more enjoyable [30,31]. Conversely, other authors found that students preferred conventional cadaveric dissection over virtual ones [32]. In general, acceptance of the ATV appeared to increase over time, suggesting that innovative technologies and teaching methods require familiarity, training, and structured implementation [4]. Our findings show that more students considered the ATV useful for reviewing anatomical knowledge, compared to the number of students that found it useful to acquire new anatomical knowledge. This support the idea that such technology should complement, rather than replace, other teaching methods within the anatomical curriculum. Feedback regarding ease of use of the ATV was less positive. On one hand, this may reflect the fact that the ATV is a relatively new product still undergoing refinement. On the other hand, both educators and students need time to become familiar with the tool and develop proficiency in its use.

Regardless, most of the students agreed or strongly agreed that they would use the ATV during their individual study time. The more enjoyable and stimulating a tool is perceived to be, the more likely students are to invest time engaging with it, ultimately increasing study duration.

Long-term retention was not evaluated in the present study, representing a limitation. Since learning outcomes were assessed immediately after the intervention, it is not possible to determine whether the ATV supports improved memory consolidation or long-term understanding compared with traditional methods. Although this was not an objective of this study, future research should incorporate delayed post-tests to assess long-term retention.

Further research is needed to clarify the role and potential of modern technologies in anatomical education, particularly in veterinary medicine. Expanding knowledge and experience in this field will allow educators to develop protocols and guidelines to standardize the use of these technologies in the anatomy curriculum. At present, ATV can only be partially integrated into the veterinary anatomy curriculum due to the limited number of animal species available. It can be used for both self-directed study and tutor-guided practical sessions. We believe that the growing body of research on the ATV will provide additional and valuable insights into how this technology can be more effectively implemented on a broader scale. The pedagogical role of the ATV could be further expanded through its structured integration into modern teaching approaches. In particular, the tool shows strong potential for use within flipped classroom models, in which students explore 3D anatomical materials independently before class, allowing in-person time to be dedicated to problem-solving. This method has been shown to be promising in enhancing students’ satisfaction in undergraduate healthcare courses and improving academic performance [33]. Similarly, the ATV can serve as an effective component of blended learning by combining digital anatomy exploration with traditional lectures and hands-on laboratory activities to provide a more flexible and diversified learning environment. Furthermore, the table could also support team-based learning (TBL) by enabling small groups of students to collaboratively explore anatomical structures, test hypotheses, and discuss spatial relationships directly on the virtual model. Developing these pedagogical strategies in future curricular planning will allow the ATV to progress from a supplementary study aid to a fully integrated component of the curriculum, enriching both individual and group learning experiences.

Although our study did not include a subgroup analysis, incorporating such an approach in future investigations could be particularly valuable for identifying which innovative teaching methods are most effective or preferred by students according to their individual characteristics, as shown in previous research conducted in human medical education [34].

We hope that these findings will support the development of a more comprehensive and structured integration of the ATV into the veterinary curriculum, optimizing its educational potential.

## Figures and Tables

**Table 1:** Statements (A1–A4) presented to the students.

A1	You are familiar with the ATV
A2	You are familiar with the TDCS
A3	You are confident in your ability to correctly identify the dog’s teeth by their common name
A4	You are confident in your ability to assign the correct Triadan number to the dog’s teeth

Students answered to the statements using a five-point Likert-scale based on the degree to which they agreed with each item (1 = strongly disagree; 2 = disagree; 3 = neutral; 4 = agree; 5 = strongly agree).

**Table 2:** Quiz questions (Q) presented in the pre and post-test.

Q1–Q2	Identify the colored teeth by their common name
Q3–Q4	Assign the correct Triadan number to the colored teeth
Q5	How many quadrants divide the oral cavity according to the TDCS?
Q6	Fill in the canine dental formula 2x (I 3/_; C 1/1; P_/_; M_/3)

**Table 3:** Feedback statements presented in the post-test.

F1	ATV is useful for reviewing anatomical knowledge
F2	ATV is useful for acquiring new anatomical knowledge
F3	ATV is useful for learning the TDCS
F4	ATV is easy to use
F5	ATV stimulates me to study Veterinary Anatomy
F6	I would use the ATV during my individual study time

Students answered to the statements using a five-point Likert scale based on the degree to which they agreed to each item (1 = strongly disagree; 2 = disagree; 3 = neutral; 4 = agree; 5 = strongly agree).

**Table 4:** Answers given by the students to statements A1–A4.

Statements and Groups	1 (Strongly Disagree)	2 (Disagree)	3 (Neutral)	4 (Agree)	5 (Strongly Agree)
A1					
A	37 (78.7%)	5 (10.6%)	4 (8.5%)	1 (2.1%)	0
C	38 (90.5%)	4 (9.5%)	0	0	0
A2					
A	46 (97.9%)	0	1 (2.1%)	0	0
C	39 (92.9%)	2 (4.8%)	1 (2.4%)	0	0
A3 pre					
A	22 (46.8%)	17 (36.2%)	5 (10.6%)	3 (6.4%)	0
C	14 (33.3%)	16 (38.1%)	11 (26.2%)	1 (2.4%)	0
A3 post					
A	1 (2.1%)	6 (12.8%)	12 (25.5%)	21 (44.7%)	7 (14.9%)
C	02 (4.6%)	2 (4.8%)	18 (42.9%)	18 (42.9%)	2 (4.8%)
A4 pre					
A	40 (85.1%)	4 (8.5%)	1 (2.1%)	2 (4.3%)	0
C	35 (83.3%)	4 (9.5%)	3 (7.1%)	0	0
A4 post					
A	0	3 (6.4%)	13 (27.7%)	21 (44.7%)	10 (21.3%)
C	0	5 (11.9%)	14 (33.3%)	15 (35.7%)	8 (19%)

Statements A1–A4 were administered in the pre-test and repeated in the post-test (A3, A4), allowing for comparisons between answers from group A (Anatomage) and group C (Control). Students answered using a 5-point Likert scale, ranging from 1 (strongly disagree) to 5 (strongly agree), according to how much they identified with each statements.

**Table 5:** Answers given by the students to the feedback questions (F) after the presentation with focus on the difference between answers given by group A (Anatomage) and group C (Control).

Statements and Groups	1 (Strongly Disagree)	2 (Disagree)	3 (Neutral)	4 (Agree)	5 (Strongly Agree)
F1					
A	0	0	1 (2.1%)	22 (46.8%)	24 (51.1%)
C	0	0	5 (11.9%)	21 (50%)	16 (38.1%)
F2					
A	0	1 (2.1%)	9 (19.1%)	8 (17%)	29 (61.7%)
C	0	2 (4.8%)	7 (16.7%)	17 (40.5%)	16 (38.1%)
F3					
A	0	0	2 (4.3%)	14 (29.8%)	31 (66%)
C	0	3 (7.1%)	8 (19%)	14 (3.3%)	17 (40.5%)
F4					
A	0	5 (10.5%)	14 (29.8%)	15 (31.9%)	13 (27.7%)
C	0	5 (11.9%)	10 (23.8%)	17 (40.5%)	10 (23.8%)
F5					
A	0	0	0	19 (40.4%)	28 (59.6%)
C	0	0	4 (9.5%)	20 (47.6%)	18 (42.9%)
F6					
A	0	0	1 (2.1%)	16 (34%)	30 (63.8%)
C	0	0	5 (11.9%)	20 (47.6%)	17 (40.5%)

Students answered using a 5-point Likert scale based on how much they agreed with each of the statements (e.g., 1 = strongly disagree; 2 = disagree; 3 = neutral; 4 = agree; 5 = strongly agree). Group A: Anatomage; Group C: control.

## Data Availability

The original contributions presented in this study are included in this article. Further inquiries can be directed to the corresponding author.

## Abbreviations

The following abbreviations are used in this manuscript:

ATV	Anatomage Table Vet
TDCS	Triadan Dental Classification System
AT	Anatomage Table
